# Expression of a bacterial xylose isomerase in an industrial strain of *Saccharomyces cerevisiae*

**DOI:** 10.1186/1753-6561-8-S4-P222

**Published:** 2014-10-01

**Authors:** Beatriz Temer, Leandro Vieira Santos, Luige Armando Calderón, Gonçalo Amarante Guimarães Pereira

**Affiliations:** 1 Universidade Estadual de Campinas, UNICAMP, Campinas, SP, Brazil; 2 Universidade Estadual de Campinas, UNICAMP, Laboratório de Genômica e Expressão, LGE, Campinas, SP, Brazil

## Background

The use of lignocellulosic biomass rather than fossil fuel is an environmental sustainable alternative for bioethanol production. However, fermentation of lignocellulosic hydrolysates by *Saccharomyces cerevisiae *is not viable since this yeast cannot ferment xylose naturally. Current, several studies are being developed to introduce a pathway that allows pentose fermentation by *S. cerevisiae *[1]. The bacterium *Propionibacterium acidipropionici*, employed in many industrial processes, is able to efficiently ferment xylose using the enzyme xylose isomerase. Xylose isomerase, codified by the *XI *gene, converts xylose to xylulose [2]. This study aims to develop a yeast capable of fermenting xylose through the expression of the *P. acidipropioniciXI *gene in *S. cerevisiae*. Furthermore, the effect of the over expression of a gene that encodes a xylulokinase (*XKS1*) and the deletion of the gene that codifies an aldose reductase (*AR*) together with the expression of the *XI *gene were evaluated. These enzymes are crucial for xylose fermentation since the former converts xylulose to xylulose-5-P by the pentose phosphate pathway (PPP) and the last converts xylose into xylitol, which can alter the xylose isomerase activity [3].

## Methods

The sequence of the *XI *gene from *P. acidipropionici *was obtained from its genome recently published [4]. An industrial strain of *S. cerevisiae *derived from PE-2, A1 (haploid; *URA3*Δ), was used in this work. The *URA3*Δ was used as an auxotrophic mark to select the transformants. The strategy chosen for heterologous expression of the *XI *gene by *S. cerevisiae *was its introduction in a high copy number plasmid. This plasmid contains the *URA3 *gene and the *XI *gene was cloned with a constitutive promoter and terminator. Three strategies were used to evaluate the heterologous expression of the *XI *gene: (1) expression of the *XI *gene alone; (2) expression of the *XI *and overexpression of the *XKS1 *gene; (3) expression of the *XI *gene, overexpression of *XKS1 *and deletion of the *AR *gene.

## Results and conclusion

Tests of growth performed in a culture medium supplemented with xylose proved the great ability of *P. acidipropionici *to grow in this carbon source. Considering that the codon usage of *S. cerevisiae *is substantially different from the *P. acidipropionici *and given that this bacterium has a high GC content, an optimization of the codons from the *XI *gene was performed. By this way, the codon adaptation index (CAI), initially 0.49, raised to 0.93 after the optimization. The optimized gene was synthetized and the yeast was transformed with the *XI *cassette. Preliminary fermentation tests in medium containing xylose as carbon source showed that these yeasts were still not able to ferment xylose. Analysis of RNA samples from all lineages confirmed that the *XI *is expressed. Since the majority of the *XI *sequences are protected by patents and the gene from this bacterium is not protected, many efforts are being done to understand the reason why this bacterial gene is not functional in *S. cerevisiae*.
